# Supplementary material for: Asymmetric division triggers cell-specific gene expression through coupled capture and stabilization of a phosphatase
Source: eLife. 2015 Oct 14;4:e08145. doi: 10.7554/eLife.08145 (PMC4714977; doi:10.7554/eLife.08145)
Supplement: Figure 4—source data 1. — Cultures of indicated strains with were grown in DSM for 28 hr at 37°C followed by heat killing for 20 min at 85°C. The number of spores was determined by counting viable cells. DOI: http://dx.doi.org/10.7554/eLife.08145.011 [file elife-08145-fig4-data1.docx]

| **Strain** | **SpoIIE variant** | **Spores/ml** |  | |
| --- | --- | --- | --- | --- |
| RL3 | wildtype | 3.95 x 10^8^ |  | |
| RL5876 | SpoIIE-YFP | 3.94 x 10^8^ |  | |
| RL5877 | SpoIIE-3XFLAG | 4.07 x 10^8^ |  | |
| RL5895 | K356D | <1 |  | |
| RL5896 | S361F | 3.00 x 10^4^ |  | |
| RL5897 | C399A | 2.50 x 10^7^ |  | |
| RL5898 | C402A | 1.00 x 10^7^ |  | |
| RL5899 | C408A W409A | 3.38 x 10^7^ |  | |
| RL5900 | C446A | 1.00 x 10^7^ |  | |
| RL5901 | I538A | 7.00 x 10^5^ |  | |
| RL5902 | L646K | <1 |  | |
| RL5903 | D686A | 5 |  | |
| RL5904 | Q483A | 126 |  | |
| RL5905 | V490K | 68 |  | |
| RL5906 | D628A | <1 |  | |
| RL5907 | E639K | 6.00 x 10^2^ |  | |
| RL5908 | E675A | 3.60 x 10^4^ |  | |
| RL5909 | S699D | <1 |  | |
| RL6052 | Tag^SpoIIE^-MalFtm | 3.90 x 10^6^ |  | |
| RL6053 | MalFtm | 9.00 x 10^5^ |  | |
|  |  |  |  | |
| RL5936 | K356D T353I | 4.40 x 10^7^ |  | |
| RL5937 | T353I S361F | <2000 |  | |
| RL5938 | T353I V490K | <1 |  | |
| RL5939 | K356D V697A | 4.70 x 10^7^ |  | |
| RL5940 | S361F V697A | 2.30 x 10^8^ |  | |
| RL5941 | V490K V697A | 1.10 x 10^7^ |  | |
| RL5942 | Q483A V697A | 1.80 x 10^8^ |  | |
|  |  |  |  | |
| **Amino acid substitutions with wild type sporulation efficiency** | | | |  |

E428A, R477A, K552A, Q508A, Y529K, D537K, E539K, D493K, L479F, K448A, E509A, R350A, R350D, T353A, E750A, T416A Y417A

**Figure 4-source data 1: Sporulation efficiency of SpoIIE mutants.**  Cultures of indicated strains with were grown in DSM for 28 hours at 37ºC followed by heat killing for 20 minutes at 85ºC. The number of spores was determined by counting viable cells.
