# Supplementary material for: Asymmetric division triggers cell-specific gene expression through coupled capture and stabilization of a phosphatase
Source: eLife. 2015 Oct 14;4:e08145. doi: 10.7554/eLife.08145 (PMC4714977; doi:10.7554/eLife.08145)
Supplement: Supplementary file 1. — DOI: http://dx.doi.org/10.7554/eLife.08145.020 [file elife-08145-supp1.docx]

| **Supplementary File 1: Table of strains** | |
| --- | --- |
|  |  |
|  |  |
| ***Bacillus subtilis* strains (all strains are in the background of PY79-RL3)** | |
| **Strain #** | **Genotype** |
| RL3 | wildtype (PY79) |
| RL5874 | *spoIIE*::*kan yxiD*::*spoIIE-yfp spc amyE*::P*_spoIIE_-cfp cm* |
| RL5875 | *spoIIE*::*kan yhdGH*::P*_spoIIQ_-cfp tet* |
| RL5876 | *spoIIE*::*kan yhdGH*::P*_spoIIQ_-cfp tet amyE*::*spoIIE-yfp spc* |
| RL5877 | *spoIIE*::*kan amyE*::*spoIIE-3XFLAG spc* |
| RL5878 | *amyE*::P*_hyperspank_-spoIIE-3XFLAG* |
| RL5879 | *amyE*::P*_hyperspank_-spoIIE-3XFLAG spc ftsH*::*mls* |
| RL5880 | *amyE*::P*_hyperspank_-spoIIE-∆tag-3XFLAG spc* |
| RL5881 | *amyE*::P*_hyperspank_-tag^spoIIE^-malF-spoIIE 3XFLAG spc* |
| RL5882 | *amyE*::P*_hyperspank_-spoIIE-∆reg-3XFLAG spc* |
| RL5883 | *amyE*::P*_hyperspank_-spoIIE-∆PP2C-3XFLAG spc* |
| RL5884 | *amyE*::P*_hyperspank_-spoIIE-∆tag-3XFLAG spc ftsH*::*mls* |
| RL5885 | *amyE*::P*_hyperspank_-tag^spoIIE^-malF-spoIIE-3XFLAG spc ftsH*::*mls* |
| RL5886 | *amyE*::P*_hyperspank_-spoIIE-∆regII-3XFLAG spc ftsH*::*mls* |
| RL5887 | *amyE*::P*_hyperspank_-spoIIE-∆regIII-3XFLAG spc ftsH*::*mls* |
| RL5888 | *amyE*::P*_hyperspank_-tag^spoIIE^-malF 3XFLAG spc* |
| RL5889 | *amyE*::P*_hyperspank_-tag^spoIIE^-malF 3XFLAG spc ftsH*::*mls* |
| RL5890 | *amyE*::P*_hyperspank_-malF-3XFLAG spc* |
| RL5891 | *amyE*::P*_hyperspank_-malF-3XFLAG spc ftsH*::*mls* |
| RL5892 | *spoIIE*::*kan yhdGH*::P*_spoIIQ_-cfp (tet) amyE*::*spoIIE-∆tag-yfp spc* |
| RL5893 | *spoIIE*::*kan amyE*::P*_spoIIQ_-lacZ cm yxiD*::*spoIIE-yfp spc* |
| RL5894 | *spoIIE*::*kan amyE*::P*_spoIIQ_-lacZ cm yxiD*::*spoIIE-∆tag-yfp spc* |
| RL5895 | *spoIIE*::*kan yhdGH*::P*_spoIIQ_-cfp tet amyE*::*spoIIE-yfp K356D spc* |
| RL5896 | *spoIIE*::*kan yhdGH*::P*_spoIIQ_-cfp tet amyE*::*spoIIE-yfp S361F spc* |
| RL5897 | *spoIIE*::*kan yhdGH*::P*_spoIIQ_-cfp tet amyE*::*spoIIE-yfp C399A spc* |
| RL5898 | *spoIIE*::*kan yhdGH*::P*_spoIIQ_-cfp tet amyE*::*spoIIE-yfp C402A spc* |
| RL5899 | *spoIIE*::*kan yhdGH*::P*_spoIIQ_-cfp tet amyE*::*spoIIE-yfp C408A,W409A spc* |
| RL5900 | *spoIIE*::*kan yhdGH*::P*_spoIIQ_-cfp tet amyE*::*spoIIE-yfp C446A spc* |
| RL5901 | *spoIIE*::*kan yhdGH*::P*_spoIIQ_-cfp tet amyE*::*spoIIE-yfp I538A spc* |
| RL5902 | *spoIIE*::*kan yhdGH*::P*_spoIIQ_-cfp tet amyE*::*spoIIE-yfp L646K spc* |
| RL5903 | *spoIIE*::*kan yhdGH*::P*_spoIIQ_-cfp tet amyE*::*spoIIE-yfp D686A spc* |
| RL5904 | *spoIIE*::*kan yhdGH*::P*_spoIIQ_-cfp tet amyE*::*spoIIE-yfp Q483A spc* |
| RL5905 | *spoIIE*::*kan yhdGH*::P*_spoIIQ_-cfp tet amyE*::*spoIIE-yfp V490K spc* |
| RL5906 | *spoIIE*::*kan yhdGH*::P*_spoIIQ_-cfp tet amyE*::*spoIIE-yfp D628A spc* |
| RL5907 | *spoIIE*::*kan yhdGH*::P*_spoIIQ_-cfp tet amyE*::*spoIIE-yfp E639K spc* |
| RL5908 | *spoIIE*::*kan yhdGH*::P*_spoIIQ_-cfp tet amyE*::*spoIIE-yfp E675A spc* |
| RL5909 | *spoIIE*::*kan yhdGH*::P*_spoIIQ_-cfp tet amyE*::*spoIIE-yfp S699D spc* |
| RL5910 | *spoIIE*::*kan yhdGH*::P*_spoIIQ_-cfp tet amyE*::*spoIIE-yfp spc spoIIAC*::*mls* |
| RL5911 | *spoIIE*::*kan yhdGH*::P*_spoIIQ_-cfp tet amyE*::*spoIIE-∆tag-yfp spc* |
| RL5912 | *spoIIE*::*kan yhdGH*::P*_spoIIQ_-cfp tet amyE*::*spoIIE-∆tag-yfp K356D spc* |
| RL5913 | *spoIIE*::*kan yhdGH*::P*_spoIIQ_-cfp tet amyE*::*spoIIE-∆tag-yfp S361F spc* |
| RL5914 | *spoIIE*::*kan yhdGH*::P*_spoIIQ_-cfp tet amyE*::*spoIIE-∆tag-yfp I538A spc* |
| RL5915 | *spoIIE*::*kan yhdGH*::P*_spoIIQ_-cfp tet amyE*::*spoIIE-∆tag-yfp L646K spc* |
| RL5916 | *spoIIE*::*kan yhdGH*::P*_spoIIQ_-cfp tet amyE*::*spoIIE-∆tag-yfp D686A spc* |
| RL5917 | *spoIIE*::*phleo spoIIAC*::*kan amyE*::*spoIIE-∆tag-yfp spc* |
| RL5918 | *spoIIE*::*phleo spoIIAC*::*kan amyE*::*spoIIE-∆tag-yfp K356D,T353I spc* |
| RL5919 | *yxiD*::P*_hyperspank_ spoIIE-yfp spc amyE*::P*_xyl_-mciz cm* |
| RL5920 | *spoIIE*::*kan yxiD*::P*_hyperspank_-spoIIE-∆tag-yfp spc amyE*::P*_xyl_-mciz cm* |
| RL5921 | *spoIIE*::*kan yxiD*::P*_hyperspank_-spoIIE-∆tag-yfp K356D spc amyE*::P*_xyl_-mciz* |
| RL5922 | *spoIIE*::*kan yxiD*::P*_hyperspank_-spoIIE-∆tag-yfp K356D,T353I spc amyE*::P*_xyl_-mciz* |
| RL5923 | *trpC2 minD*::*mls divIVA*::*tet amyE*::P*_xyl_-mciZ cm yxiD*::P*_hyperspank_-spoIIE-∆tag-yfp spc* |
| RL5924 | *yxiD*::P*_hyperspank_-spoIIE-∆tag-yfp spc amyE*::P*_xyl_-mciz cm* |
| RL5925 | *spoIIE*::*kan yxiD*::*spoIIE-∆tag-yfp spc amyE*::*divIVA-FLAG cm* |
| RL5926 | *spoIIE*::*kan yxiD*::*spoIIE-∆tag-yfp spc K356D amyE*::*divIVA-FLAG cm* |
| RL5927 | *spoIIE*::*phleo lacA*::P*_xyl_-cfp-zapA mls amyE*::*pspank-spoIIE-yfp tet divICΩdivICts spc* |
| RL5928 | *spoIIE*::*phleo lacA*::P*_xyl_-cfp-zapA mls amyE*::*Pspank-spoIIE-∆tag-yfp tet divICΩdivICts spc* |
| RL5929 | *spoIIE*::*phleo spoIIAC*::*kan amyE*::*spoIIE-∆tag-yfp cm lacA*::P*_xyl_-cfp-zapA mls divIBD*::*spec* |
| RL5930 | *amyE*::*ftsAZ, cm zae-86*::*P_spank_-spoIIE-gfp kan, spc, cat spo0A*::*mls* |
| RL5931 | *amyE*:: *ftsAZ cm ylnF*::*Pspank-SpoIIA operon tet lacA*::P*_xyl_-racA mls yxiD*::P*_hyperspank_-spoIIE spc yycR*::P*_spoIIQ_-yfp phleo spo0A*::*kan* |
| RL5932 | *amyE*::P*_spoIIQ_-malF-∆tag-yfp spc* |
| RL5933 | *amyE*::P*_spoIIQ_-malF-yfp spc* |
| RL5934 | *amyE*::P*_spoIIQ_-spoIIE-malF-∆tag-3XFLAG spc* |
| RL5935 | *amyE*::P*_spoIIQ_-spoIIE-malF-3XFLAG spc* |
| RL5936 | *spoIIE*::*kan yhdGH*::P*_spoIIQ_-cfp tet amyE*::*spoIIE-yfp K356D, T353I spc* |
| RL5937 | *spoIIE*::*kan yhdGH*::P*_spoIIQ_-cfp tet amyE*::*spoIIE-yfp T353I, S361F spc* |
| RL5938 | *spoIIE*::*kan yhdGH*::P*_spoIIQ_-cfp tet amyE*::*spoIIE-yfp T353I, V490K spc* |
| RL5939 | *spoIIE*::*kan yhdGH*::P*_spoIIQ_-cfp tet amyE*::*spoIIE-yfp K356D, V697A spc* |
| RL5940 | *spoIIE*::*kan yhdGH*::P*_spoIIQ_-cfp tet amyE*::*spoIIE-yfp S361F, V697A spc* |
| RL5941 | *spoIIE*::*kan yhdGH*::P*_spoIIQ_-cfp tet amyE*::*spoIIE-yfp V490K, V697A spc* |
| RL5942 | *spoIIE*::*kan yhdGH*::P*_spoIIQ_-cfp tet amyE*::*spoIIE-yfp Q483A, V697A spc* |
| RL5943 | *spoIIE*::*Kan yhdGH*::P*_spoIIQ_-cfp tet amyE*::*SpoIIE-yfp K356D T353I spc spoIIAC*::*mls* |
| RL5944 | *spoIIE*::*Kan yhdGH*::P*_spoIIQ_-cfp tet amyE*::*SpoIIE-yfp K356D V697A spc spoIIAC*::*mls* |
| RL6040 | *amyE*::P*_hyperspank_-spoIIE spc* |
| RL6041 | *amyE*::P*_hyperspank_-spoIIE spc ftsH::mls* |
| RL6042 | *spoIIE::kan amyE::spoIIE-yfp spc lacA::*P*_xyl_-cfp-zapA mls* |
| RL6043 | *spoIIE::kan amyE::spoIIE-yfp spc lacA::*P*_xyl_-cfp-zapA mls ylnF::*P*_spank_-malFtm-mneptune tet* |
| RL6044 | *spoIIE::phleo amyE::spoIIE-gfp spc ylnF::*P*_spank_-malFtm-mneptune tet* |
| RL6045 | *spoIIE::phleo amyE::spoIIE-yfp spc spoIIAC::kan racA::tet* |
| RL6046 | *spoIIIE-36 spoIIE::kan ylnF::spoIIE-yfp spc spoIIAC::mls* |
| RL6052 | *spoIIE*::*kan yhdGH*::P*_spoIIQ_-cfp tet amyE*::*tag^spoIIE^-malFtm-spoIIE-yfp spc* |
| RL6053 | *spoIIE*::*kan yhdGH*::P*_spoIIQ_-cfp tet amyE*:: *malFtm-spoIIE-yfp spc* |
|  |  |
| ***E. coli* strains** | |
| RL5945 | *BL21 (DE3) pET23a H6-sumo-spoIIE 320-827* |
| RL5946 | *BL21 (DE3) pET23a H6-sumo-spoIIE 320-827 V697A* |
| RL5947 | *BL21 (DE3) pET23a H6-sumo-spoIIE 345-827* |
| RL5948 | *BL21 (DE3) pET23a H6-sumo-spoIIE 358-827* |
| RL5949 | *BL21 (DE3) pET23a H6-sumo-spoIIE 320-827 K356D* |
| RL5950 | *BL21 (DE3) pET23a H6-sumo-spoIIE 320-827 K356D, T353I* |
| RL6047 | *BL21 (DE3) pET23a H6-sumo-spoIIE 320-827 Q483A* |
| RL6048 | *BL21 (DE3) pET23a H6-sumo-spoIIE 320-827 D628A* |
| RL6049 | *BL21 (DE3) pET23a H6-sumo-spoIIE 320-827 E639K* |
| RL6050 | *BL21 (DE3) pET23a H6-sumo-spoIIE 320-827 S361F* |
| RK6051 | *BL21 (DE3) pET23a H6-sumo-spoIIE 320-827 D686A* |

All *Bacillus subtilis* strains are in the PY79 background. All point mutations were introduced by quickchange mutagenesis, and in all cases *∆tag-spoIIE* removed amino acids 11-38 from *spoIIE* by ITA. Details of strain construction follow:

*spoIIE*::*kan* the entire *spoIIE* open reading frame was knocked out by long flanking homology PCR using the kanamycin cassette from pDG780 (Guérout-Fleury et al. 1995).

*amyE*::P*_spoIIE_-cfp cm* was made by amplifying the first 200 basepairs upstream of *spoIIE* and fusing it to a codon optimized monomeric *cfp* (Doan et al. 2005) inserted into pDG1661 digested with BamHI, BlpI by isothermal assembly.

*yhdGH*::P*_spoIIQ_-cfp tet* was from (Besprozvannaya et al. 2013)

*amyE*::*spoIIE-yfp spc* contains the spoIIE open reading frame fused to *yfp* and containing the promoter region from 216nt upstream. Cloned into pDG1728 digested with BamHI and BlpI.

*yxid*::*spoIIE* constructs were made by transforming RL4932 (Amy Camp) containing the tn917 minitransposon at the *yxid* locus with plasmid DNA for *amyE*::*spoIIE-yfp* constructs.

*amyE*::*spoIIE-3XFLAG* contains the *spoIIE* open reading frame fused to 3XFLAG (Asp-Tyr-Lys-Asp-His-Asp-Gly-Asp-Tyr-Lys-Asp-His-Asp-IIe-Asp-Tyr-Lys-Asp-Asp-Asp-Asp-Lys) (Sigma Aldrich) and containing the promoter region from 216nt upstream of *spoIIE*. Cloned into pDG1728 with BamHI and BlpI.

*amyE*::P*_hyperspank_-spoIIE-3XFLAG* contains the *spoIIE* open reading frame inserted by ITA to pDR111a digested with HindIII, and NheI (does not preserve HindIII site). Contains 19bp upstream of *spoIIE* to include the native ribosome binding site.

*ftsH*::*mls* was made by long flanking homology PCR to remove the entire *ftsH* ORF. *mls* was amplified from RL3545 (Camp & Losick 2008)

*amyE*::P*_hyperspank_-tag^spoIIE^-malF-SpoIIE-3XFLAG* contains the first two transmembrane domains of *E. coli malF* (King et al. 1999) fused to 3XFLAG and the *spoIIE* ORF beginning at amino acid 320 (note that unlike in King et al., the entire 38 amino acid N-terminal cytoplasmic tail of SpoIIE was fused to malF). The construct was inserted by ITA to pDR111a digested with HindIII, and NheI (does not preserve HindIII site). Contains 19bp upstream of *spoIIE* to include the native ribosome binding site.

*amyE*::P*_hyperspank_-spoIIE-∆reg-3XFLAG* removed amino acids 320-568 of SpoIIE and was inserted to pDR111a digested with HindIII and NheI by ITA (does not preserve HindIII site). Contains 19bp upstream of *spoIIE* to include the native ribosome binding site.

*amyE*::P*_hyperspank_-spoIIE-∆PP2C-3XFLAG* removed amino acids 590-827 of SpoIE and was inserted to pDR111a digested with HindIII and NheI by ITA (does not preserve HindIII site). Contains 19bp upstream of *spoIIE* to include the native ribosome binding site.

*amyE*::P*_hyperspank_-tag^spoIIE^-malF-3XFLAG* had the entire 38 amino acid N-terminal cytoplasmic tail of SpoIIE fused to the first two transmembrane domains of *E. coli malF* (King et al. 1999) fused to 3XFLAG. The construct was inserted to pDR111a digested with HindIII, and NheI by ITA (does not preserve HindIII site). Contains 19bp upstream of *spoIIE* to include the native ribosome binding site.

*amyE*::P*_hyperspank_-malF-∆tag-3XFLAG* had the first ten amino acids of SpoIIE fused to the first two transmembrane domains of *E. coli malF* (King et al. 1999) fused to 3XFLAG. The construct was inserted to pDR111a digested with HindIII, and NheI by ITA (does not preserve HindIII site). Contains 19bp upstream of *spoIIE* to include the native ribosome binding site.

*amyE*::P*_spoIIQ_-lacZ* is a translational fusion of P*_spoIIQ_* to lacZ (Camp & Losick 2009).

*spoIIAC*::*mls* (Eichenberger et al. 2001) gift of P. Stragier RL1275

*spoIIE*::*phleo* (Arigoni et al. 1999)

*spoIIAC*::*kan* (Price & Losick 1999) gift of P. Stragier RL1265

*amyE*::P*_xyl_-mciz* (Handler et al. 2008)

*trpC2 amyE- minD*::*erm divIVA*::*tet* (Edwards & Errington 1997)

*amyE*::*divIVA-FLAG cm* (Eswaramoorthy et al. 2014)

*lacA-*P*_xyl_-cfp-zapA mls* Used ITA to make *cfp-zapA* under xylose inducible promoter in pAX01 digested with BamHI, SacII. ACATAAGGAGGAACTACT was appended before the start of codon optimized monomeric cfp (Doan et al. 2005).

*amyE*::*Pspank-spoIIE-yfp tet* The vector was constructed by digestion of pDR110a with EcoRI, AsiSI to remove *spcR* which was replaced by the *tetR* construct from pDG1514 digested with EcoRI, PvuI. *spoIIE-yfp* including 19bp upstream of the start codon were inserted by ITA to vector digested with HindIII and SphI. *∆tag-spoIIE* removed amino acids 11-38 from *spoIIE* by ITA.

*divICΩdivICts spc* (Levin & Losick 1994)

*amyE*:: *∆tag-spoIIE-yfp cm* contains the *spoIIE* open reading frame fused to *yfp* and containing the promoter region from 216nt upstream. Cloned into pDG1661 with BamHI and BlpI. ∆tag spoIIE removed amino acids 11-38 from *spoIIE* by ITA.

*divIB*::*spc* is a campbell integration of an internal Sau3A fragment of the gene (From +146 to +335) built by Freddy Gueiros-Filho. RL2224

*amyE*::*ftsAZ, cm zae-86*::P*_spank_-spoIIE-gfp kan* (Carniol et al. 2005)

*spo0A*::*mls* (Ireton et al. 1993)

*ylnF*::P*_spank_-SpoIIA operon tet* The P*_spank_ tet* vector was constructed by digestion of pDR110a with EcoRI, AsiSI to remove *spcR* which was replaced by the *tetR* construct from pDG1514 digested with EcoRI, PvuI. It was constructed by PCR amplification of the *spoIIA* operon adding HindIII and SpHI sites. Includes 26bp upstream to include the native RBS.

*lacA*::P*_xyl_-racA mls racA* including 22bp upstream to include the native RBS was inserted to pAX01 digested with BamHI and SacII.

*yxiD*::P*_hyperspank_-spoIIE spc* (Carniol et al. 2005) RL3466

*yycR*::P*_spoIIQ_-yfp phleo* (Besprozvannaya et al. 2013)

*spo0A*::*kan* (Chu et al. 2006) RL4620

*amyE*::P*_spoIIQ_-malF-yfp spc* The promoter of *spoIIQ* (200bp upstream of the *spoIIQ* ORF) was fused to *malF-yfp* and inserted to pDG1728 digested with BamHI and BlpI.

*amyE*::P*_spoIIQ_-tag^spoIIE^-malF-yfp spc* The promoter of *spoIIQ* (200bp upstream of the *spoIIQ* ORF) was fused to *tag^spoIIE^-malF-yfp* and inserted to pDG1728 digested with BamHI and BlpI.

*amyE*::P*_spoIIQ_-malF-3XFLAG spc* The promoter of *spoIIQ* (200bp upstream of the *spoIIQ* ORF) was fused to *malF-3XFLAG* and inserted to pDG1728 digested with BamHI and BlpI.

*amyE*::P*_spoIIQ_-tag^spoIIE^-malF-3XFLAG spc* The promoter of *spoIIQ* (200bp upstream of the *spoIIQ* ORF) was fused to *tag^spoIIE^-malF-yfp* and inserted to pDG1728 digested with BamHI, BlpI.

*BL21 (DE3) pET23a H6-sumo-spoIIE 320-827* pET23a with 6H-sumo was digested with NotI, AgeI, and *spoIIE* from amino-acids 320-827 amplified with restriction sites appended was inserted by ligation.

**References**

Arigoni, F. et al., 1999. The SpoIIE phosphatase, the sporulation septum and the establishment of forespore-specific transcription in Bacillus subtilis: a reassessment. *Molecular microbiology*, 31(5), pp.1407–1415.

Besprozvannaya, M. et al., 2013. SpoIIIE protein achieves directional DNA translocation through allosteric regulation of ATPase activity by an accessory domain. *The Journal of biological chemistry*, 288(40), pp.28962–28974.

Camp, A.H. & Losick, R., 2009. A feeding tube model for activation of a cell-specific transcription factor during sporulation in Bacillus subtilis. *Genes & development*, 23(8), pp.1014–1024.

Camp, A.H. & Losick, R., 2008. A novel pathway of intercellular signalling in Bacillus subtilis involves a protein with similarity to a component of type III secretion channels. *Molecular microbiology*, 69(2), pp.402–417.

Carniol, K. et al., 2005. Genetic dissection of the sporulation protein SpoIIE and its role in asymmetric division in Bacillus subtilis. *Journal of bacteriology*, 187(10), pp.3511–3520.

Chu, F. et al., 2006. Targets of the master regulator of biofilm formation in Bacillus subtilis. *Molecular microbiology*, 59(4), pp.1216–1228.

Doan, T., Marquis, K.A. & Rudner, D.Z., 2005. Subcellular localization of a sporulation membrane protein is achieved through a network of interactions along and across the septum. *Molecular microbiology*, 55(6), pp.1767–1781.

Edwards, D.H. & Errington, J., 1997. The Bacillus subtilis DivIVA protein targets to the division septum and controls the site specificity of cell division. *Molecular microbiology*, 24(5), pp.905–915.

Eichenberger, P., Fawcett, P. & Losick, R., 2001. A three-protein inhibitor of polar septation during sporulation in Bacillus subtilis. *Molecular microbiology*, 42(5), pp.1147–1162.

Eswaramoorthy, P. et al., 2014. Asymmetric Division and Differential Gene Expression during a Bacterial Developmental Program Requires DivIVA. *PLoS Genetics*, 10(8), p.e1004526.

Guérout-Fleury, A.M. et al., 1995. Antibiotic-resistance cassettes for Bacillus subtilis. *Gene*, 167(1-2), pp.335–336.

Handler, A.A., Lim, J.E. & Losick, R., 2008. Peptide inhibitor of cytokinesis during sporulation in Bacillus subtilis. *Molecular microbiology*, 68(3), pp.588–599.

Ireton, K. et al., 1993. Integration of multiple developmental signals in Bacillus subtilis through the Spo0A transcription factor. *Genes & development*, 7(2), pp.283–294.

King, N. et al., 1999. Septation, dephosphorylation, and the activation of sigmaF during sporulation in Bacillus subtilis. *Genes & development*, 13(9), pp.1156–1167.

Levin, P.A. & Losick, R., 1994. Characterization of a cell division gene from Bacillus subtilis that is required for vegetative and sporulation septum formation. *Journal of bacteriology*, 176(5), pp.1451–1459.

Price, K.D. & Losick, R., 1999. A four-dimensional view of assembly of a morphogenetic protein during sporulation in Bacillus subtilis. *Journal of bacteriology*, 181(3), pp.781–790.
